# *In vivo* regeneration of renal vessels post whole decellularized kidneys transplantation

**DOI:** 10.18632/oncotarget.6321

**Published:** 2015-11-13

**Authors:** JianSe Zhang, ZhiBin Wang, KeZhi Lin, YaLing Yu, LiNa Zhao, TingGang Chu, LiZhi Wu, Ali Alkhawaji, MiaoZhong Li, YingKuan Shao, Ting Li, XinFa Lou, ShiXin Chen, MaoLin Tang, Jin Mei

**Affiliations:** ^1^ Institute of Bioscaffold Transplantation and Immunology, Wenzhou Medical University, Wenzhou, China; ^2^ Anatomy Department, Wenzhou Medical University, Wenzhou, China; ^3^ Department of Orthopedics, The Second Affiliated Hospital of Wenzhou Medical University, Wenzhou, China; ^4^ Department of Hand and Foot Surgery, Luqiao Hospital of Enze Medical Center, Taizhou, China; ^5^ Department of Anatomy, King Saud bin Abdulaziz University for Health Sciences, Riyadh, Saudi Arabia; ^6^ Institute of Neuroscience, Wenzhou Medical University, Wenzhou, China

**Keywords:** renal vessels, scaffold, microsurgery, orthotopic transplantation, renal regeneration, Pathology Section

## Abstract

Nearly 50 million patients in China live with end-stage renal disease (ESRD), and only about 4000 patients may receive kidney transplantation. The purpose of this study was to investigate regeneration of renal vessels post whole decellularized kidneys transplantation *in vivo*. We decellularized kidneys of donor rats by perfusing a detergent through the abdominal aorta, yielding feasible extracellular matrix, confirmed for acellularity before transplantation. Based on the concept of using the body as a bioreactor, we orthotopically transplanted the kidney and ureter scaffolds in recipient rats, and found the regeneration of vessels including artery and vein in the renal sinus following a spontaneous recanalization. Although the findings only represent an initial step toward the ultimate goal of the generation of fully functional kidneys *in vivo*, these findings suggest that the body itself, as the bioreactor, is a viable strategy for kidney regeneration.

## INTRODUCTION

Chronic kidney disease (CKD) is a global health issue. Nearly 120 million patients live with CKD in China alone [1]. Although ESRD can be managed with dialysis, up to date there is no simple therapy for this condition. Dialysis partially replaces the filtration properties of the kidney, but does not address the loss of homeostatic and endocrine functions [2-4]. Alternative to dialysis, which could holistically restore homeostatic renal functions, is kidney transplantation. However, the supply of donor kidneys is drastically insufficient to meet the demand, which in turn, has caused prolonging waiting lists. Less than one percent of ESRD patients in China may receive kidney transplantation [1], however, lifelong immunosuppression is required to reduces the risk of chronic rejection [5, 6]. A potential solution to address these challenges is the application of organ engineering, which could be used to develop functional organ replacements in a timely manner.

Organ engineering has proven to be capable for bioartificial organ constructions by using decellularized scaffolds [7, 8]. The harvested donor organs are decellularized to remove the cells, and thereafter repopulated with cells. *In vitro* perfusion is required to establish the circulation of nutrients and gases, and is achieved by the use of a bioreactor and suitable perfusion medium, potentially an oxygen carrier, and - for some organs - a biophysical stimulator [9, 10]. Notable advances have been made in the development of organ engineering by using scaffold-based designs. Organ engineering has been successfully reported in a variety of organs including the heart [11], trachea [12, 13], liver [8], and lung [14-16]. Although this *in vitro* regenerative approach overpasses previously published methods, it requires high costs and it is hard to simulate the internal natural and physiological conditions. A recently published report about tracheal tissue engineering *in vivo* [16] found that the body of the animals could be used as a potential bioreactor when transplants are in a normotopic position. However, as for parenchymatous organs, with more complex composition, it is unknown whether this concept could achieve satisfactory results.

**Figure 1:**
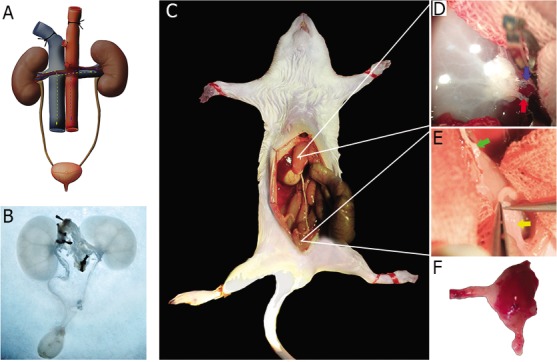
Schema of using autologous bio-reactor to rebuild the left kidney of the decellularized scaffold **A.** The decellularization process in kidney and ureter. Abdominal aorta cannulated for infusion of detergent solutions. Other vessels except the renal artery were blocked. **B.** Decellularized kidney and ureter appear white-translucent in color. **C.** Acellular matrix was implanted orthotopically into recipient rat with vascular anastomosis and bladder to ureter anastomosis. **D.**, **E.** The topography of implanted matrix. The red arrow shows the renal artery, the blue arrow shows the renal vein, the green arrow shows the ureter and the yellow arrow shows the bladder. **F.** The implanted scaffolds were then harvested for further observation 1 to 8 weeks post implantation.

Our previous study has revealed that transplanting of decellularized kidney scaffolds is able to partially restore the renal functions in rat [17], We thus hypothesized that the body has the ability to act as a natural bioreactor for kidney regeneration. The steps of our approach are summarized in Figure 1. The objectives of this study were to investigate the biological properties of the remaining ECM; further to transplant the unseeded kidney scaffolds orthotopically into healthy SD rats and to investigate how the renal vessels were regenerated successfully following continues host immune responses in decellularized kidney scaffolds.

## RESULTS

### Characterization of decellularized kidney scaffolds

**Figure 2:**
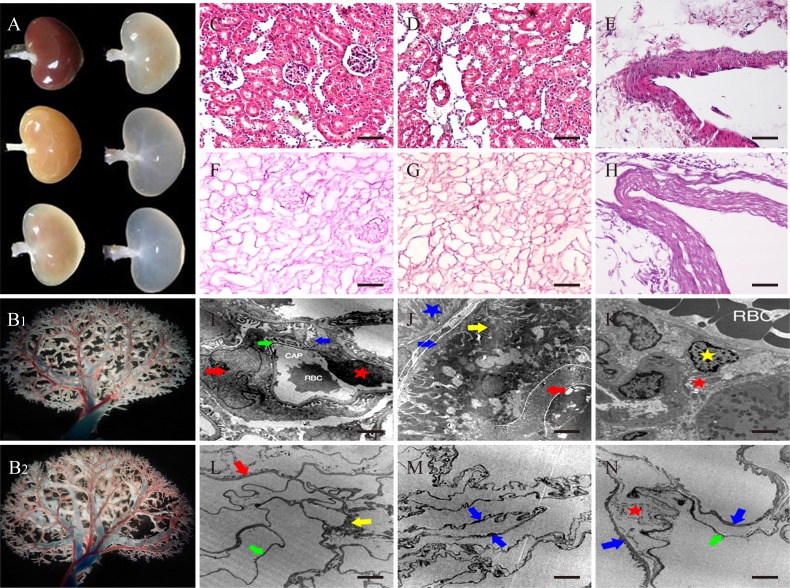
Morphology of decellularized kidney scaffolds **A.** Gross color changes of the decellularized kidneys. **B1.** Vascular corrosion casting of native kidney. **B2.** Vascular corrosion casting of decellularized kidney. All showed three dimensional structure of small arteries and veins. **C.**-**E.** H&E stain of native rat kidney. **F.**-**H.** H&E stain of acellular kidney matrix. **I.**-**K.**TEM of native kidney. **L.**-**N.** TEM of Decellularized kidney. **I.** The red arrow shows podocyte, and blue arrow was its foot process. Green arrow was basement-membrane. The red star was vascular endothelial cell (VEC). **J.** The red arrow shows microvilli and the yellow arrow shows the fold of plasma membrane. The blue arrow was the membrane of tubule and the green star was the nucleus. The blue star was mitochondria. **K.** The yellow star was the interstitial cell and the red star was collagen fibers. **L.** The red arrow shows the membrane of Bowman capsule. The yellow arrow was mesangium, and the green arrow was capillary membrane. **M.** The two blue arrows both were the membrane of tubule. **N.** The two blue arrows both were the membrane of tubule. The green arrow was capillary membrane and the red star was collagen fibers. (C-H) Scale bars =100μm. (I-N) Scale bars =2.5μm.

**Figure 3:**
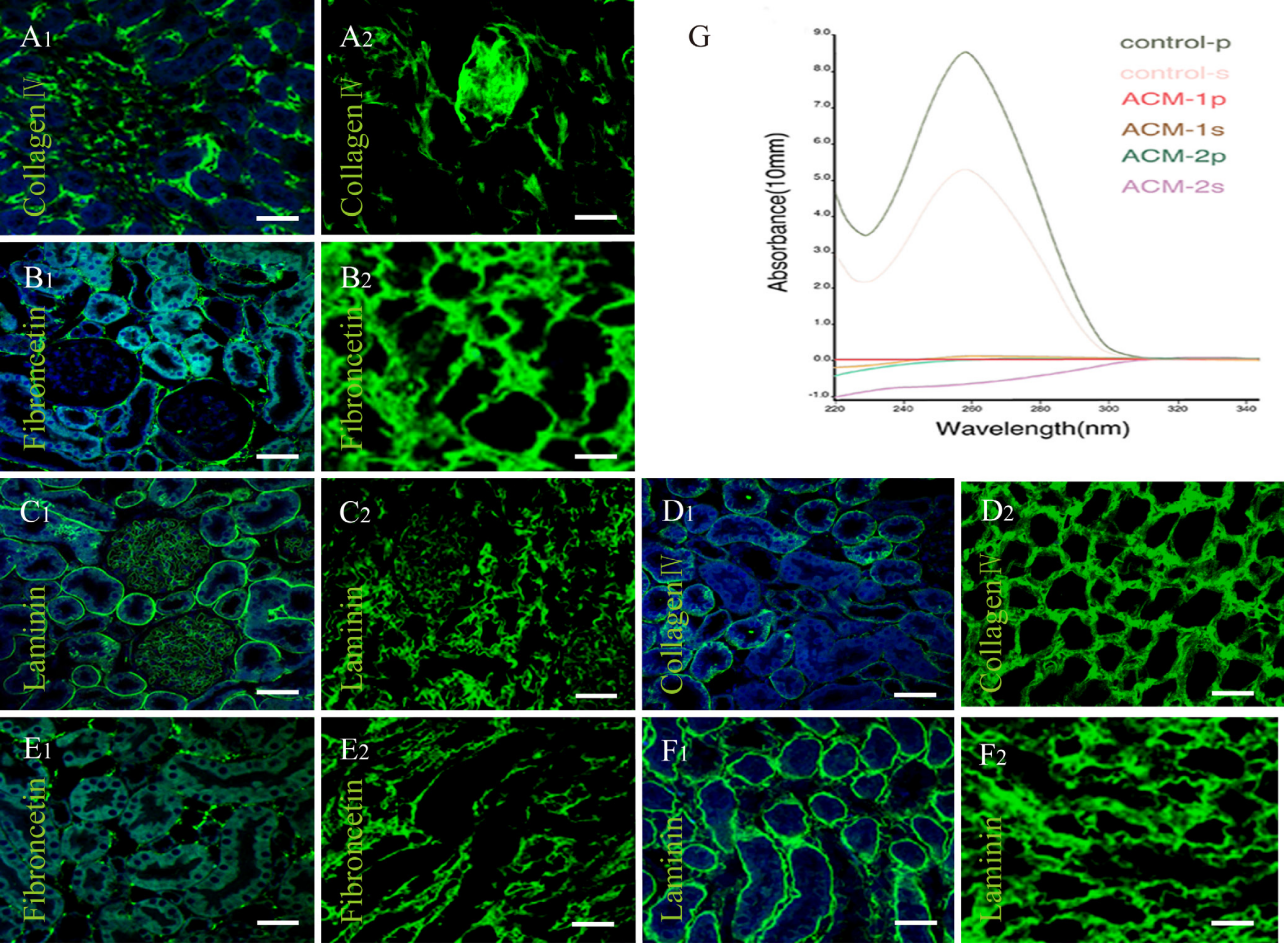
DNA analysis and Immunofluorescence of decellularized kidney scaffolds **A.**-**F.** Immunofluorescence of Collagen IV, Fibronectin (FN) and Laminin(LN). (A1-C1) Cortex of native kidney. (D1-F1) Medulla of native kidney. (A2-C2) Cortex of decellularized kidney. (D2-F2) Medulla of decellularized kidney. (A-F) Scale bars =20μm. In all panels, cell nuclei stain was blue with DAPI, while fluorescent immunohistochemical staining for specific markers appears green. These indicated the intact kidney architecture and matrix proteins were retained and undisturbed, while cells and nuclear material was removed compared with the native. **G.** Curve diagram of DNA content in native (control) kidney and renal acellular matrix (ACM). The number P represents renal cortex; the S represents renal medulla. This revealed a significant decrease in the total amount of DNA in decellularized kidneys.

After the decellularization of Sprague Dawley (SD) kidney, the scaffolds maintained the three dimensional structure, and became homogeneously translucent due to the removal of the cellular contents (Figure 1B, and Figure 2A). The quantitative analysis and agarose gel electrophoresis results of the tissue genomic DNA (gDNA) content revealed a significant decrease in the total amount of DNA in the decellularized kidneys. UV spectrophotometer assay showed that about 97% of gDNA was removed in the decellularized kidney compared to the native kidney (Figure 3G). The mean residual Sodium dodecyl sulfate (SDS) concentration in the decellularized kidney scaffolds was safe as we reported previously [17]. Vascular corrosion casting of the whole decellularized kidney showed that the three dimensional structure of the vasculature was preserved and intact compared with the native (Figure 2B1, 2B2).

Microscopic examination of hematoxylin and eosin (H&E) stained decellularized kidney specimens revealed that the integrity of the glomerular capsule, tubules and vessels was intact and cells were all removed compared with the native (Figure 2C-2H). In addition, transmission electron microscopy (TEM) assay showed that the decellularized kiney scaffolds were clear of any cellular/nuclear material compared with the native kidney, but the basement membrane extracellular matrix of the renal glomeruli, the renal tubules were kept intact (Figure 2I-2N), suggesting the potential to support regenerative ability. Further immunofluorescence assay counterstained with DAPI (4′, 6 -diamidino-2-phenylindole) indicated the intact kidney matrix proteins, such as Laminin (LN), Collagen IV and Fibronectin (FN). These proteins were retained and undisturbed, while cells and nuclear material was removed compared with the native tissue (Figure 3A-3F). Taken together, the decellularized scaffolds prepared in our study were acellular, maintaining the continuous extracellular matrix and the intact three dimensional vascular structures.

### Endothelial progenitor cells (EPCs) culture and biocompatibility of acellular scaffold *in vitro*

**Figure 4:**
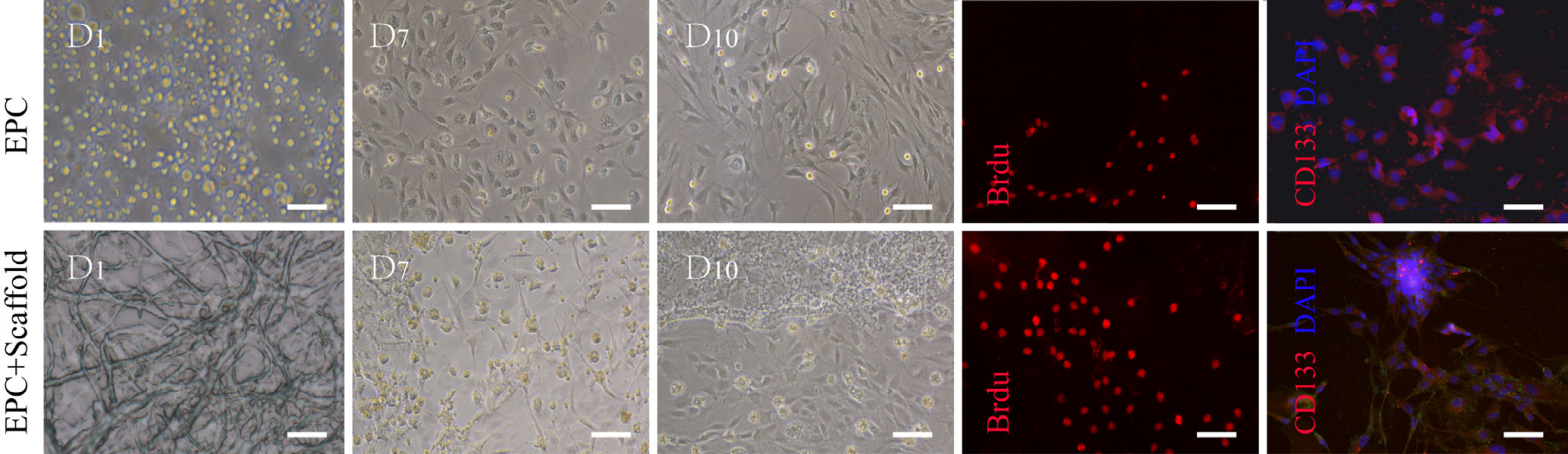
Characteristics of EPCs and biocompatibility of acellular scaffold *in vitro* The morphology of cells cultured from bone marrow mononuclear cells displayed round and spindle-shaped on 1-10 days. The EPCs were observed to adhere to the acellular scaffolds and grow in excellent condition. Immunofluorescence revealed the expression of BrdU and the surface biomarkers CD133 in both group on 3 and 10 days separately, suggesting the acellular scaffolds have the ability to induce the adhesion and proliferation of the cultured cells. Cell nuclei are stained with DAPI (blue). Scale bars = 20μm.

Mononuclear cells (MNC) were isolated and harvested from SD rat's femur bone marrow by density gradient centrifugation. Then these cells were cultured with EGM-2 MV BulletKit *in vitro.* After cell culture, the attached cells, namely EPCs, were precursor cells of vascular endothelial cells. The data of culture and co-culture with the scaffolds showed EPCs changed its morphology from round to spindle in shape, and formed networks within the scaffolds. Immunofluorescence revealed the expression of CD133, a surface biomarkers for vascular endothelial cells, on day 10. These cells were clearly seen and adhered to the acellular scaffolds. The number of EPCs labeled with bromodeoxyuridine (BrdU) displayed an apparent increase within the scaffolds, 3 days after the co-culture (Figure 4), suggesting that the acellular scaffolds have the ability to induce the adhesion and proliferation of the cultured EPCs.

### Evaluation of the cellular regeneration in the explanted grafts

**Figure 5:**
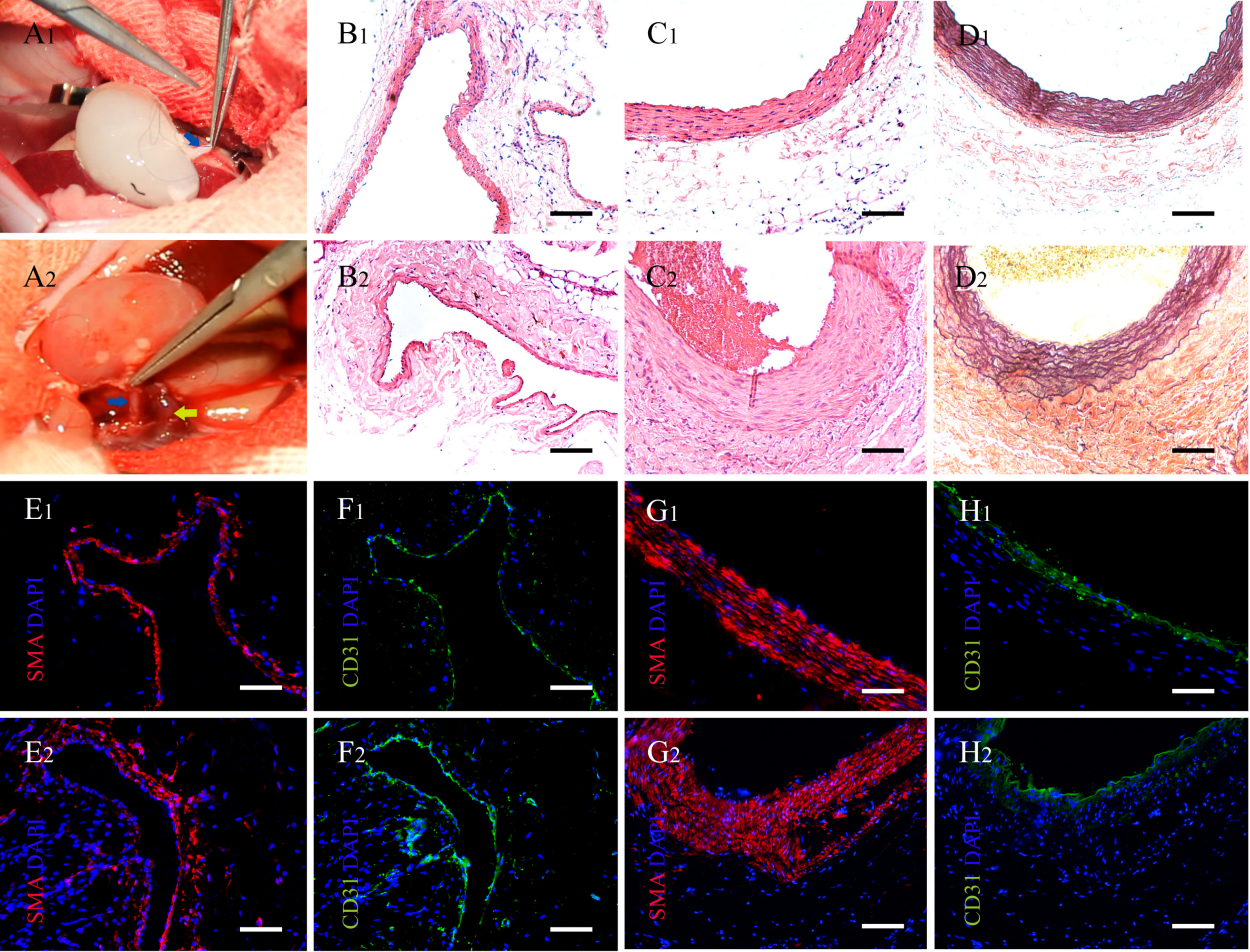
Implantation of acellular kidney and characteristics of renal vessels in the explanted scaffolds **A1.** The decellularized kidney scaffolds were implanted orthotopically into the recipient. Vascular anastomosis was performed. The blue arrow shows the renal artery. **A2.** The perfusion of blood into the scaffolds after vascular anastomosis. The blue arrow shows the renal artery and the yellow arrow shows the renal vein. **B.**-**H.** The vascular structures on two weeks were regenerated and rearranged compared to the native in the renal sinus. (B1-H1) The vessels of renal sinus in native kidney. (B2-H2) The vessels of renal sinus in explanted scaffolds post-implantation two weeks. (B) H&E stains of the vein. (C) H&E stains of the artery. (D) Weigert stains of the artery. (E-H) Immunofluorescence of the CD31 (green) and the SMA (red). (E, F) Vein. (G, H) Artery. Cell nuclei stain was blue with DAPI. (B-H) Scale bars =100μm.

**Figure 6:**
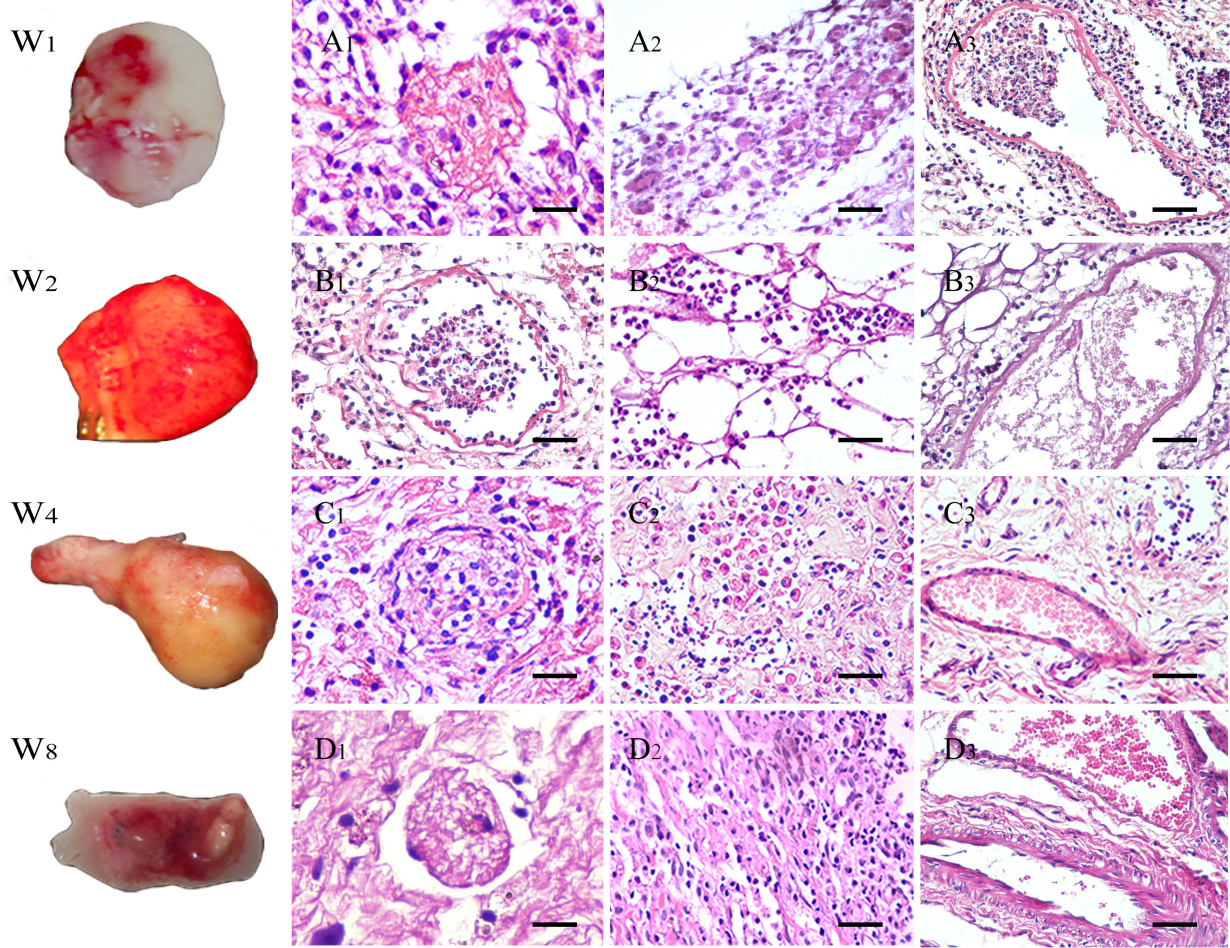
General appearance and H&E stain of explanted scaffolds after 1 to 8 weeks (W1-8) Gross appearance of explanted kidney scaffolds. **A.**-**D.** H&E stains of glomeruli, tubule and vessels. (A1-D1) Glomeruli gradually became scared from infection. (A2-D2) The structures of kidney tubules were replaced with other tissue and cells. (A3-D3) The Inflammatory cell infiltration reduced significantly and some neovascularization were observed in the grafts. The thickness of the vascular walls was increased, apparently in the 8th week, especially of the arteries in the explanted grafts. (A1-D2) Scale bars =25μm; (A3-D3) Scale bars =50μm.

To evaluate the regeneration potential in the scaffolds, fifty scaffolds were orthopedically transplanted into fifty recipient rats (Figure 5A1, 5A2). The majority of the recipients, forty-four rats, survived through the implantation procedure and six died. Out of the forty-four rats, three died one day after the operation, two died one week later and two developed hematuria two weeks later. To examine the morphologic structures of the explanted grafts, the grafts were analyzed at various post-surgical time points. Gross observation showed that the grafts atrophied gradually and lost its original shape with the span of time (Figure 6). H&E sections were then prepared for analyzing the distribution of blood vessel in the regenerated site. The blood clots were observed in the renal vessels at the anastomosis sites in twelve rats; two died on the first day and ten were sacrificed at one or two weeks after the surgery (five for each). However, no blood clots were observed in all samples four weeks after the operation.

One and two weeks post-implantation, the kidney scaffolds were infiltrated by inflammatory cells, especially around the kidney glomerulus and tubules. The morphology of glomeruli, which were infiltrated less cells, was intact but developed fibrosis at 4 weeks (Figure 6A1-6D1). The number of inflammatory cells decreased and the type changed during the development of tissues. The morphology of graft changed and the structures of kidney tubules were replaced of other tissue and cells (Figure 6A1-6D2). The regeneration of the renal vessels was observed in five out of nineteen samples. The thickness of the vascular walls was increased, apparently in the 8th week, especially of the arteries in the explanted grafts (Figure 5B-5C and Figure 6A3-6D3). And the weirget staining revealed that the integrity of the elastic fibers in vessels was intact. (Figure 5D). Further immunofluorescence assay counterstained with DAPI indicated the endothelial and smooth muscle layers of the vessels in the renal sinus were regenerated visibly as post-implantation time progressed (Figure 5E2-5H2).

## DISCUSSION

The de novo reconstruction of the kidney is more challenging than the regeneration of many other tissues due to the complexity and diversity of the components and structures of the kidney. However, recent studies indicated that the kidney has the intrinsic capacity to regenerate [18]. The decellularized scaffolds retains the natural vascular network and components, which are conserved between species [19-22]. Over the past few years, a remarkable progress in the regenerative medicine has been made. Song JJ et al [7] used an artificial bioreactor to regenerate partially functioning kidneys by repopulating the kidney scaffolds with human umbilical venous endothelial and rat neonatal kidney cells. While Orlando et al [21]confirmed that vivo implantation of porcine kidney scaffolds is technically feasible, although the vascular structures were occluded by thrombosis. Serpooshan et al [23] demonstrated that the formation of new networks of interconnected blood vessels within myocardial infarction site *in vivo* is promoted by an acellular collagen patch with speciﬁc biomechanical properties. Our previous study showed that decellularized kidney scaffolds can be used to mediate kidney regeneration [17]. Based on this, we hypothesized that the body itself has the ability to act as a natural bioreactor for kidneys.

In our experiment, the rat kidneys are efficiently decellularized to create whole renal ECM scaffolds in a relatively short time, with perfusable vessels, and intact glomerular and tubular compartments. The decellularized kidney scaffold was non-cytotoxic and maintained some kinds of cytokines, proved to promote cell adhesion and proliferation *in vitro*. Then the prepared acellular scaffolds are implanted orthotopically to preserve the critical role of the internal environment. Some neovascularization were observed inside the grafts. The grafts were infiltrated by a large number of inflammatory cells in the initial stage, and decreased gradually. The scaffolds lost its original shape with the extension of the time, but still there was the emergence of new blood vessels inside. Some researchers have indicated that fibroblasts could arise through different ways; from endothelial cells *via* the process of endothelial-to-mesenchymal transition, from epithelial cells *via* epithelial-to-mesenchymal transition or from the bone marrow during kidney fibrosis [24]. In addition, the endothelial cells, elastic fibers, and smooth muscle layers of the vessels were observed. However the mechanism of regeneration in vessels and the process of cellular transformation were poorly understood in the grafts. It may be the suppression of inflammatory action facilitates the cell transformation and regulates diverse biological processes [25, 26] in a period post implantation to motivate the regeneration.

Our study suggests that the body can be used as a bioreactor, since the internal environment of the living organisms has the potential physiological capability that could help in regeneration, and ensures protective stability for the tissues and organs. The internal environment is a good source of nutrients, various growth factors, and provides a media for interactions between cell-cell and cell-matrix. The internal environment can also support cell survival and function by promoting cells differentiation and rearrangement within the acellular graft. The natural bioreactors would provide an opportunity for auto-repopulation, so this could be used in the future to develop fully functional organs. In our study, the implantation was challenging because it required microsurgical skills, however it is technically possible to implant the scaffolds orthotopically in small animals.

The cell-ECM interaction and 3D microenvironment plays a role in cell morphology, migration, proliferation, differentiation and formation of tissue-like structures. As such, there might be some molecular mechanism(s) that can trigger the release of cytokines and similar signals that promote cell migration proliferation and differentiation. The appropriately prepared matrix will retain signals to trigger site-specific differentiation of pluripotent precursor cells, and thereby launch a cascade of matrix-cell, cell-cell and cell-matrix events culminating in cycles of organ differentiation and remodeling of the original scaffolds [27].

We observed the regeneration of some renal vessels, but the regeneration of glomeruli and tubules have not been observed. The implantation of decellularized whole kidney scaffolds, without repopulation, is technically feasible in rats. Although the findings only represent an initial step toward the ultimate goal of the generation of fully functional kidneys *in vivo*, these findings suggest that the body itself, as the bioreactor, is a viable strategy for kidney regeneration.

## MATERIALS AND METHODS

### Preparation of decellularized scaffolds

In total, ninety (twenty-five for scaffold donors + fifty recipients + fifteen for analysis) healthy SD rats at the age of about two months, weighing 200-250g were used in this experiment. The animals were provided by Laboratory Animal Center of Wenzhou Medical University, Zhejiang Province, P. R. China. The experiment was approved by the administration of Wenzhou Medical University and conducted in accordance with ethical guidelines for the use and care of animals. Twenty-five rats were anesthetized with 5% chloral hydrate (0.6 ml/100g) via intraperitoneal injection. The abdominal cavity was opened though a ventral midline incision from the pubis to the xyphoid process. A 24 G cannula was then inserted into the abdominal aorta to allow anterograde perfusion using a peristaltic pump (YX1515X-A; Baoding Longer Precision Pump Co., China). Considering that the cannulation can easily damage the renal vessel, we chose to cannulate the abdominal aorta instead. Phosphate buffer saline (PBS, pH 7.4) 500ml containing 100 units of heparin was perfused to remove all the blood in the kidney at a rate of 8mL/min using a peristaltic pump at 37°C, followed by 1000ml 0.1% (v/v) Triton X-100, 200ml deionized water and 2000ml 0.8% (v/v) SDS. Fifty decellularized kidney scaffolds, produced from the previous step, were gently washed with 5000 ml deionized water containing 1% (v/v) penicillin/streptomycin and heparin to remove all of the cellular debris and chemical residues. The decellularized kidney scaffolds were sterilized using a medical electron linear accelerator X - Gray (2300 C/D) at a dose of 2 KC Gray for 4 minutes and stored in sterile PBS containing penicillin-streptomycin and heparin at 4°C for less than 7 days.

### Assessment of genomic DNA content and SDS residue levels

The residue level of genomic DNA was assessed in decellularized kidney scaffolds and compared with native kidneys. Tissue samples were digested with 20mg/ml proteinase K at 65°C for 2-3 h. The genomic DNA was extracted using the Universal Genomic DNA Extraction Kit Ver.3.0 (Takara Bio Inc., Japan). The quantity and purity of the DNA was assayed using NanoDrop 2000 UV-Vis Spectrophotometer (Thermo Scientific, USA). Then, the DNA samples were run on 1% agarose gel with ethidium bromide and observed under Ultaviolet (UV) light. Considering that SDS is toxic to cells, UV-VIS was used to assess SDS residue level in the scaffolds.

### Transmission electron microscope (TEM)

TEM was used to examine the ECM in decellularized kidney scaffolds and compared with native kidneys. Samples were harvested and ﬁxed with 2.5% (v/v) glutaraldehyde in 0.1 M sodium cacodylate buffer overnight at 4°C, then ﬁxed with 1% osmium tetroxide for 1 h at 37°C. Subsequently, the specimens were dehydrated with a series of acetone solutions of increasing concentration, beginning with 70%, then 80%, 90% and 100% absolute acetone, infiltrated with epon resin and baked overnight at 65°C. Following this, the hardened blocks were trimmed into semi-thin slices and quickly examined using a light microscope. Selected slices were made into ultrathin sections of 80 nm and stained using 2% uranyl acetate and lead citrate. All samples were examined on a Hitachi TEM (H7500; Japan) at 70kV. Images were taken using a gatan 830 high resolution CCD digital camera.

### Histology and immunofluorescence

For histological evaluation, samples were fixed in 10% paraformaldehyde at 4°C overnight, then dehydrated in a graded series of ethanol and permeabilized in dimethyl benzene and embedded in paraffin wax. After the paraffin embedding, 4-μm thick sections were consecutively cut, and stained with H&E to analyze the morphology and microstructure. For DNA content evaluation, the sections were then counterstained with DAPI. Then, the sections were observed with immunofluorescence to detect the basement membrane proteins, which are composed of ECM components including laminin, collagen IV, and ﬁbronectin.

For immunofluorescence, tissue sections were deparaffinized, and rehydrated, and antigen retrieval was performed. Sections were then blocked with 10% fetal bovine serum (FBS) for 30 minutes at room temperature. Primary antibodies were applied and left to incubate overnight at 4°C, followed by 488- or 594-conjugated species-specific secondary antibody (Chemicon, USA) at 1:400 dilutions for 2 hour at 37°C. Lastly the sections were rinsed in PBS for 45 minutes, and stained with DAPI. Slides were observed using an Olympus fluorescent microscope and images were taken using Olympus soft image viewer.

### Vascular corrosion casting of kidney scaffolds

To determine the three-dimensional microvasculature in the scaffolds, catheterization of the inferior vena cava and abdominal aorta was performed, followed by injection of 1~2ml acetone depending on the kidney size. Then, 10% Acrylonitrile Butadiene Styrene (ABS) Sudan solvent mixture 5ml was rapidly injected through abdominal aorta. Meanwhile, 10% ABS blue pigment mixture 10ml was perfused into the inferior vena cava. The samples were cooled under running water and corroded in 50% hydrochloric acid for 1-3d. The morphology and distribution of vasculature was observed under stereomicroscope and images were taken with Olympus soft image viewer.

### EPCs culture and co-culture with the decellularized scaffolds *in vitro*

To further confirm the decellularized scaffolds ability to support cell attachment and growth, Mononuclear cells (MNC) were isolated and harvested from rats femur bone marrow by density gradient centrifugation. Then these cells were cultured with EGM-2 MV BulletKit *in vitro*. After 3 day, unadhered cells were removed s from the culture, and the medium was changed every 3-4 days. After decellularization and sterilization of the scaffolds, they were cut into 100um thick slices and co-cultured with 1×10^5^ EPCs in a media and was allowed to equilibrate with 5% CO2 atmosphere at 37°C for 10 days. Then the EPC cells and its proliferations were visualized, 3 days post culturing with BrdU and 10 days pot culturing with by CD133, using a Nikon AZ-100 fluorescence microscope for characterization.

### Implantation of decellularized scaffolds

To investigate the study hypothesis, we implanted 50 decellularized scaffolds into 50 rats. (Supplementary Video). Under a surgical microscope (XTS-4A, ZTGX, Jiangsu, China), the left kidney and ureter of the recipient were removed and heparin (100 units/kg) was injected intravenously through the inferior vena cava to prevent blood clots. Then the renal artery and vein of the decellularized kidney scaffold were microsurgically anastomosed to the left renal vessels of the recipient using 10-0 PROLENE monofilament sutures with 4 stitches for the artery and 8 stitches for the vein (Figure 5A1) Shortly after the removal of the vascular clamp, the blood flowed into the decellularized renal vessels and the implanted kidney scaffolds turn red without blood leakage (Figure 5A2). Then, an incision to the recipient bladder was made, and the decellularized donor ureter was anastomosed to connect the recipient bladder to the donor kidney.

Then the recipient rat was observed for 30 minutes to ensure there was no blood leakage or clot formation. The abdominal wall was then closed layer by layer using 4-0 PROLENE monofilament suture. After surgery, all animals were allowed to unlimited access to both rat chow and water containing penicillin and streptomycin (100u/ml) and subcutaneous injection with heparin 5000 U for more than one week.

### Evaluation of the explanted grafts

To evaluate the regeneration of scaffolds *in vivo*, groups the rats were sacrificed at 1, 2, 4 and 8 week(s) post-implantation respectively. Then sections from the explanted grafts were stained with H&E and analyzed for morphology and microstructure.

## SUPPLEMENTARY VIDEO


